# The Impact of Colonoscopy Quality Control Table on Adenoma Detection Rates

**DOI:** 10.1155/2016/2548109

**Published:** 2016-06-02

**Authors:** Bin Deng, Jiehua Zhi, Yaosheng Chen, Lanyu Liang, Jian Wu, Xuefen Gao, Weiming Xiao, Yanbing Ding

**Affiliations:** ^1^Department of Gastroenterology, Yangzhou No. 1 People's Hospital, Jiangsu 225001, China; ^2^Department of Emergency, Yangzhou No. 1 People's Hospital, Jiangsu 225001, China

## Abstract

*Objective*. This study aims to investigate the effects of reporting colonoscopy findings and the regular review of outcomes on adenoma detection rates.* Methods*. Patients who underwent colonoscopy from August 2013 to February 2014 were selected as the intervention group. The preintervention group included patients who underwent colonoscopy from January 2013 to July 2013, in which the procedure sheet for this group of patients was not accomplished. The primary outcome was adenoma detection rate (ADR), and secondary outcomes included the success rate of intubation and withdrawal time.* Results*. This study included 2,467 cases: 1,302 cases in the intervention group and 1,165 cases in the preintervention group. There was no significant difference in demographic characteristics between the two groups. In the intervention group, withdrawal time of colonoscopy was longer (*P* < 0.01), and the success rate of intubation (92.5% versus 89.1%, *P* < 0.05) and detection rate of polyps (32.6% versus 27.6%, *P* < 0.05) and adenomas (20.0% versus 16.1%, *P* < 0.05) were higher. Significantly high detection rates for proximal adenomas, flat adenomas, and adenomas with a diameter <5 mm were observed in the intervention group (all *P* < 0.01).* Conclusion*. The reporting and review of procedure details help to improve quality indicators of colonoscopy.

## 1. Introduction

The incidence of colon cancer continues to decline in the United States, which is most likely due to the implementation of screening procedures and increased use of colonoscopy; and the effectiveness of colonoscopy in preventing colorectal cancer (CRC) is largely based on the detection and removal of adenomatous polyps [1, 2]. However, studies have suggested that high variations in the quality of colonoscopy among different endoscopists are reflected in surrogate measures such as adenoma detection, cecal intubation rates, withdrawal times, and incidence of complications. Adenoma detection rate (ADR) is an independent risk factor for predicting interval CRC [3]. Corley et al. reported that each 1% increase in ADR was associated with a 3% decrease in the risk of interval CRC and a 5% decrease in the risk of fatal interval cancers [4]. Several effective interventions have improved the quality of colonoscopy including prolonged withdrawal, as well as the use of chromoendoscopy, closed-circuit television, and so on [5–8]. However, few studies have investigated the effects of the data reporting and reviewing strategies on colonoscopy [9].

The American Society for Gastrointestinal Endoscopy recently introduced a quality recognition program that recognizes endoscopy units that have implemented quality improvement and monitoring processes as an integral part of their operations. A quarterly report card has been shown to improve colonoscopy quality indicators. The quarterly report card is a practical intervention and may serve as a model for quality improvement programs in different patient and physician groups [9].

We hypothesize that the reporting and regular review of procedure details would improve the quality of colonoscopy. This study aims to evaluate the effect of this intensive and regular monitoring and feedback program on colonoscopy quality indicators such as ADRs, intubation success, and withdrawal time.

## 2. Materials and Methods

This prospective study was approved by Institutional Ethics Committee of Yangzhou No. 1 People's Hospital.

### 2.1. Study Subjects

All patients who underwent colonoscopy in Yangzhou No. 1 People's Hospital from 1 January 2013 to 28 February 2014 were screened.

### 2.2. Inclusion Criteria

Patients aged 18–75 years undergoing colonoscopies were included in this study.

### 2.3. Exclusion Criteria

Patients were excluded based on the following: (1) colorectal cancer in first- or second-degree relatives; (2) history of colon cancer, colectomy, familial adenomatous polyposis, or inflammatory bowel diseases; (3) severe intestinal stenosis or obstruction; (4) coagulation disorders; (5) pregnancy; (6) colonoscopy examinations with inadequate bowel cleanliness, as defined by a Boston Bowel Preparation Scale (BBPS) score of <6.

### 2.4. Colonoscopy Procedure

Colonoscopy was performed using a standard colonoscope (Olympus CF Q260), but procedures were not performed with any special equipment such as chromoendoscopy, narrow-band imaging (NBI), or cap-assisted colonoscopy in this study.

### 2.5. Methods

Two groups were formed in this study: preintervention group and intervention group. In the intervention group, colonoscopy was performed, and the quality control table was accomplished; meanwhile in the preintervention group, colonoscopy was performed, but the quality control table was not accomplished. The new quality monitoring program based on the “Report and Review” strategy commenced on 1 August 2013. Subsequently, physicians who performed endoscopic procedures at the Medical Center completed the procedure sheet (see the Appendix). The procedure sheet contains the patient's basic profile, colonoscopy quality indicators including the documentation of bowel preparation quality, cecal intubation, withdrawal time and ADR, details of anesthesia, insertion and withdrawal time, complications, and the name and professional title of the physician. Patients who underwent colonoscopy from August 2013 to February 2014 were selected as the intervention group. Twelve professional colonoscopists at our Endoscopy Center were responsible for the examination in the preintervention and intervention groups and table filling. There was no statistical difference in endoscopic operation cases performed by each physician between the two groups. Data were collected regularly and statistically analyzed. All physicians publicly received their own data once a month and compared their performances with the performance of the rest of the group. The two groups were compared in terms of the characteristics of polyps or adenomas including the size, site, shape, number and pathological results, withdrawal time, success rate of intubation, and complications. Withdrawal time was based on adequately cleansed colons with no lesions requiring biopsy or polypectomy by subtracting the biopsy or polypectomy time.

### 2.6. Data Collection and Definitions

Data collection and definitions are as follows:

1. High-risk adenomas were defined by any of the following conditions: the number of adenomas being ≥3, the diameter of adenomas being ≥1 cm, or villous adenoma or high-grade intraepithelial neoplasia [10].
2. Sites of the adenomas were confirmed by colon anatomy and the insertion length of the colonoscope during withdrawal procedures. Proximal colon included the transverse colon, ascending colon, ileocecal valve, and cecum.
3. Detection rate of polyps or adenomas refers to the proportion of patients with polyps or adenomas in all subjects.
4. BBPS is a valid and reliable tool that provides a more granular evaluation of bowel cleanliness, and this tool has the ability to preserve differences in bowel preparation quality among different segments of the colon [11].

### 2.7. Statistical Analysis

Measurement data are presented as mean ± standard deviation, and comparison among means was performed by *t*-test. Count data were presented as the rate or constituent ratio, and comparison of rates was performed by Chi-square test with statistical significance set at *P* < 0.05. Wilcoxon rank-sum tests were performed to compare these two groups for differences in the number of adenomas per subject. Fisher's exact tests and Mann-Whitney *U* test were performed as indicated. Data was analyzed using Statistical Package for the Social Sciences (SPSS) version 18.0.

## 3. Results

**Figure 1:**
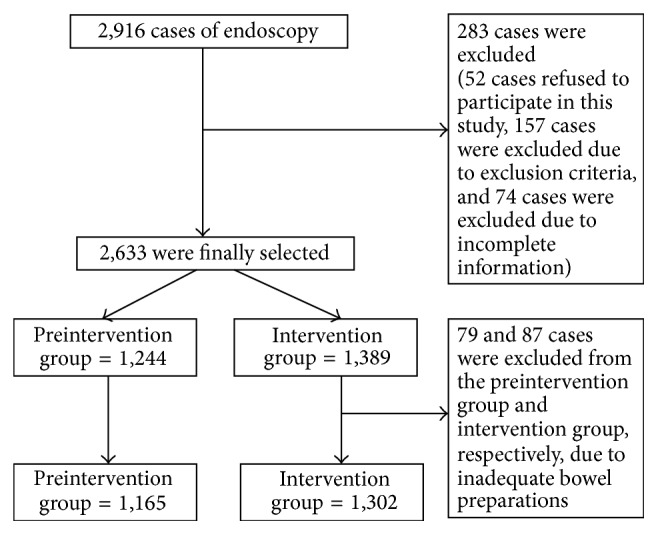
Flow chart of inclusion and exclusion criteria.

A total of 2,916 patients who underwent colonoscopy at our hospital were enrolled in this 14-month study (January 2013 to February 2014). The final selection included 2,633 eligible patients after excluding 283 cases due to exclusion criteria (157), denial to participate in the study (52), and incomplete data (74). However, 79 cases in the preintervention group and 87 cases in the intervention group were further excluded from this statistical analysis due to poor bowel preparation. Therefore, 2,467 cases were statistically analyzed including 1,165 cases in the preintervention group (for the first seven months) and 1,302 cases in the intervention group (for the following seven months), as shown in Figure 1. Colonoscopy was performed by six senior colonoscopists (>1,000 cases) and six junior colonoscopists (<1,000 cases) in both time periods. All colonoscopists have performed over 300 cases of colonoscopy. Patient demographics and colonoscopy indications are summarized in Table 1. There was no statistical difference in age, gender, or colonoscopy indications between the two groups (*P* > 0.05), as shown in Table 1.

Withdrawal time was longer (8.27 ± 3.21 minutes versus 6.38 ± 3.19 minutes, *P* < 0.001) and the success rate of intubation was higher (92.5% versus 89.1%, *P* < 0.05) in the intervention group, compared with the preintervention group. A total of 1,503 adenomas were found in 2,467 patients, including 951 adenomas in the intervention group and 552 adenomas in the preintervention group. For each patient, the number of adenomas was 0.73 in the intervention group and 0.47 in the preintervention group (*P* < 0.05). Detection rates of polyps (32.6% versus 27.6%, *P* = 0.007) and adenomas (20.0% versus 16.1%, *P* = 0.014) were higher in the intervention group. Subgroup analysis revealed no statistical significant differences in the detection rate of high-risk adenomas between the two groups (*P* > 0.05). A statistically significant higher detection rate of proximal adenoma, flat adenomas, and adenomas with a diameter <5 mm was observed in the intervention group (Table 2).

Data was divided into two subgroups in order to evaluate the impact of the endoscopist's experience on the outcome, senior colonoscopists (>1,000) and junior colonoscopists (<1,000), as shown in Table 3. In both subgroups, withdrawal time was significantly higher in the intervention group compared to the preintervention group, while there was a statistically significant difference in ADR and intubation time among junior colonoscopists but not among senior colonoscopists (Table 3).

## 4. Discussion

Detection rates of adenomas and cecal intubation are validated measures of colonoscopy performance quality [4, 12]. Our results indicate that completion of the procedure sheet and feedback improved ADR, especially for small adenomas with a diameter <5 mm and flat adenomas, but not the detection rate of high-degree adenomas. In addition, this strategy increases the average number of adenomas, which greatly reduced the risk of interval stage colorectal cancer. According to the report by Robertson et al., 52% of interval stage colorectal cancers were caused by missed adenomas during diagnosis [13]. Our study revealed a higher completion (cecal intubation) rate in the intervention group, compared with the preintervention group (92.5% versus 89.1%,  *P* = 0.004). Some studies have indicated that the high success rate of intubation was associated with high ADR and the low incidence rate of right-sided colon cancer [14]. Baxter et al. [12] reported that patients who underwent colonoscopy performed by an endoscopist with a high completion (cecal intubation) rate were less likely to reveal postcolonoscopy colorectal cancer, and this effect was observed for both distal and proximal CRCs. These colonoscopy findings are operator dependent. Interventions that are aimed at improving the quality of colonoscopy performance include specialized training and self-auditing [15]. For example, Coe et al. revealed that ADR increased to 47% for those with training compared to 35% in controls [16]. Madhoun and Tierney [8] reported that video recording of colonoscopies was associated with a nonsignificant increase in ADR and a significant increase in hyperplastic polyp detection rate. In our study, we regularly checked the quality of colonoscopy and statistically analyzed the performance of colonoscopists. We also encouraged colonoscopists to share their experiences. Several studies [15, 16] have shown that education can improve the detection rate of adenomas. Our study results are consistent with recently published studies. Kahi et al. reported that a quarterly report card on colonoscopy quality was associated with improved quality indicators. In this study, 928 cases were divided into two groups: the preintervention group (336 cases) and intervention group (592 cases). A significantly higher ADR (53.9% versus 44.7%, *P* = 0.013) and success rate of intubation (98.1% versus 95.6%, *P* = 0.027) were observed in the intervention group. In addition, most adenomas were distributed at the proximal colon and were serrated, and there was no significant difference in high-degree adenomas [9].

Our study differs from other similar studies, wherein we included more endoscopic examinations and colonoscopists over a longer study period. In the past 14 months, a total of 2,916 cases of endoscopy were recorded, and our data is more comprehensive. There was no statistical difference in the effects of single- or two-handed operation, anesthesia, and bowel preparation quality between the two groups. We publicly released the quality control results and reported these during monthly meetings to motivate colonoscopists to enhance their competency levels. Junior colonoscopists revealed significantly higher enhancement in detection rates and significantly prolonged withdrawal time, indicating that they should be prioritized for future monitoring and improvement. In our study, ADR rate was lower than that reported by Western studies [9]; and the reason was due to the low incidence of adenomas in our population, as reported by other Chinese authors [17].

Several limitations in our study warrant further discussion. First, the present study was performed at a single tertiary care referral center. Multicenter trials with larger groups of patients and colonoscopists are needed to confirm these findings. Second, we did not identify the long-term effects of our monitoring and feedback. Additionally, findings in this study may be affected by reporting bias, since the procedure sheet was accomplished by the endoscopist (Hawthorne effect) [18].

In conclusion, the reporting and regular monitoring of details of each procedure, as well as feedback, increase ADR. This strategy can be applied across different medical settings. It is easy to comprehend and is acceptable to most colonoscopists.

## Figures and Tables

**Table 1:** Comparison of patient demographics and colonoscopy indices.

Demographic characteristics	Preintervention group (*n* = 1,165)	Intervention group (*n* = 1,302)	*P*
Male (%)	578 (49.6%)	678 (52.1%)	0.222
Mean age (years)	55.81 ± 12.97	54.94 ± 13.71	0.133
BMI	23.73 ± 3.14	23.56 ± 3.29	0.192
Educational level			0.123
Junior middle school or lower	501 (43.0%)	520 (39.9%)	
Senior middle school or higher	664 (57.0%)	782 (60.1%)	
Rate of anesthesia	1,054 (90.5%)	1,200 (92.2%)	0.135
Rate of single-handed colonoscopy	1004 (86.2%)	1143 (87.8%)	0.235
Indications for colonoscopy			0.071
Screening	132 (11.3%)	172 (13.2%)	
Diagnosis	776 (66.6%)	810 (62.2%)	
Reexamination	257 (22.1%)	320 (24.6%)	

**Table 2:** Colonoscopy indices and adenoma classification (cases, %).

	Preintervention group (*n* = 1,165)	Intervention group (*n* = 1,302)	*P*
Withdrawal time (minutes)	6.38 ± 3.19	8.27 ± 3.21	*<*0.001
Success rate of intubation	1,038 (89.1%)	1,204 (92.5%)	0.004
Adenomas per case	552 (0.47)	951 (0.73)	0.006
Detection rate of polyps	321 (27.6%)	424 (32.6%)	0.007
Detection rate of adenomas	188 (16.1%)	260 (20.0%)	0.014
Diameter < 5 mm	62 (33.3%)	123 (47.3%)	0.002
Flat adenomas	57 (30.3%)	113 (43.5%)	0.005
High-risk adenomas	35 (18.6%)	44 (16.9%)	0.642
Proximal adenomas	65 (34.6%)	120 (46.4%)	0.014

**Table 3:** Comparison of colonoscopy indices between senior and junior colonoscopists.

Professional title indexes of colonoscopy	Preintervention group (*n* = 1,165)	Intervention group (*n* = 1,302)	*P*
Junior withdrawal time	6.24 ± 2.06	8.84 ± 2.49	*<*0.001
Detection rate of adenomas	59/510 (11.57%)	90/540 (16.7%)	0.018
Success rate of intubation	443/510 (86.9%)	494/540 (91.5%)	0.016
Senior withdrawal time	6.66 ± 2.69	7.92 ± 2.74	0.001
Detection rate of adenomas	106/655 (16.2%)	146/762 (19.2%)	0.144
Success rate of intubation	595/655 (90.8%)	710/762 (93.2%)	0.210

## Appendix

### Procedure Recording Sheet

Date of filling: Month— Day—

*(1) Basic Information*

Name:
Gender:
  (1) Male
  (2) Female
Age (years):
Tel.:
Hospitalized?
  (1) No
  (2) Yes
Inpatient No.:
Height (meters):
Weight (kg):
Educational background:
  (1) Illiteracy
  (2) Primary school
  (3) Middle school or technical secondary school
  (4) College
Chief complaints:

*Reasons for Colonoscopy*

(1) Treatments of polyps
(2) Physical examinations
(3) Reexaminations
(4) Rise in tumor markers
(5) Symptoms of colorectal cancer (please select one or more):
  (1) Abdominal pain
  (2) Diarrhea
  (3) Constipation
  (4) Alternating between diarrhea and constipation
  (5) Increased defecation frequency
  (6) Change in bowel habit
  (7) Melena
  (8) Blood in stool
  (9) Anemia
  (10) Emaciation
  (11) Thinner stool
  (12) Incomplete emptying of bowel
  (13) Positive OB
  (14) Others

*(2) Patient History and Comorbidity*

Date of last colonoscopy:
  Digestive tract polyp
    (1) YES
    (2) NO
  Digestive tract neoplasms
    (1) YES
    (2) NO
  Abdominal surgery
    (1) YES
    (2) NO
  Family history
    (1) YES, specification:
    (2) NO
  Family history of CRC in 1st-degree relative
    Number of family members:
    Age of index family member(s) who had CRC:
  Family history of adenoma in 1st-degree relative
    Family history of inherited syndrome
      FAP:
      HNPCC:

*(3) Sedation Colonoscopy *

(1) YES
(2) NO

*(4) Extent of Examination*

Actual extent of examination (anatomic segment: cecum, ascending colon, hepatic flexure, etc.)
If cecum is not reached, provide reason:
Method of documentation: that is, photo of ileocecal valve and/or appendiceal orifice (if possible, where equipment available); name and landmarks
Time of examination:
  The following times points should be recorded:
    Time when scope was inserted into rectum
    Time when withdrawal from cecum was started
    Time when endoscope was withdrawn from patient

*(5) Type of Preparation and Dosage*

Quality (bowel preparation quality based on the Boston Bowel Preparation Scale):
  Left-sidedness colon (descending colon, sigmoid colon, and rectum)
    (1) 0 point
    (2) 1 point
    (3) 2 points
    (4) 3 points
  Midpiece colon (hepatic flexure, transverse colon, and splenic flexure)
    (1) 0 point
    (2) 1 point
    (3) 2 points
    (4) 3 points
  Right-sidedness colon (ascending colon, ileocecal valve and cecum)
    (1) 0 point
    (2) 1 point
    (3) 2 points
    (4) 3 points
(0) Unprepared colon segment with mucosa could not be seen due to solid stool, which could not be cleared.
(1) Portion of the mucosa of the colon segment is seen, but other areas of the colon segment could not be seen well due to staining, residual stool and/or opaque liquid.
(2) Minor amount of residual staining, small fragments of stool and/or opaque liquid, but the mucosa of the colon segment could be seen well.
(3) Entire mucosa of the colon segment could be seen well with no residual staining, small fragments of stool or opaque liquid.

Each region of the colon receives a “segment score” from 0 to 3, and these segment scores are aggregated for a total BBPS score ranging from 0 to 9. Therefore, the maximum BBPS score for a perfectly clean colon without any residual liquid is 9, and the minimum BBPS score for an unprepared colon is 0.

*(6) Technical Performance*

Examination not technically difficult
Examination difficult
Comments could include:
  Patient discomfort
  Looping

*(7) Haplo-Hand Enteroscope*

(1) YES
(2) NO

*(8) Check Consequence*

*(9) Operator Name*—+—

  Professional Title:

*(10) Enteroscope Complication*

(1) YES, specification:
(2) NO

*(11) Complication of Anesthesia*

(1) YES, specification:
(2) NO
